# Recent HbA1c Values and Mortality Risk in Type 2 Diabetes. Population-Based Case-Control Study

**DOI:** 10.1371/journal.pone.0068008

**Published:** 2013-07-05

**Authors:** Jennifer Nicholas, Judith Charlton, Alex Dregan, Martin C. Gulliford

**Affiliations:** 1 London School of Hygiene and Tropical Medicine, Department of Medical Statistics, London, United Kingdom; 2 King’s College London, NIHR Biomedical Research Centre at Guy’s and St Thomas’ NHS Foundation Trust and King’s College London, Department of Primary Care and Public Health Sciences, London, United Kingdom; Charité University Medicine Berlin, Germany

## Abstract

This study aimed to evaluate mortality within 365 days of HbA1c values of <6.5% or >9.0% in participants with clinical type 2 diabetes mellitus. A matched nested case-control study was implemented, within a cohort of participants with type 2 diabetes from 2000 to 2008. Conditional logistic regression was used to model the odds ratio for mortality adjusting for comorbidity and drug utilisation. There were 97,450 participants with type 2 diabetes; 16,585 cases that died during follow up were matched to 16,585 controls. The most recent HbA1c value was <6.5% (48 mmol/mol) for 22.2% of cases and 24.2% of controls, the HbA1c was >9.0% for 9.0% of cases and 7.7% of controls. In a complete case analysis, the adjusted odds ratio (AOR) for mortality associated with most recent HbA1c <6.5% was 1.31 (95% confidence interval (CI): 1.21,1.42). After multiple imputation of missing HbA1c values the AOR was 1.20 (CI: 1.12,1.28). The complete case analysis gave an AOR for HbA1c >9.0% of 1.51 (CI: 1.33, 1.70), in the multiple imputation analysis this was 1.29 (1.17,1.41). The risk associated with HbA1c <6.5% was age dependent. In the multiple imputation analysis the AOR was 1.53 (CI: 0.84 to 2.79) at age<55 years but 1.04 (CI: 0.92, 1.17) at age 85 years and over. In non-randomised data, values of HbA1c that are either <6.5% or >9.0% may be associated with increased mortality within one year in clinical type 2 diabetes. Relative risks may be higher at younger ages.

## Introduction

Diabetes represents a major public health concern and efforts to control hyperglycaemia are an important element of the management of patients with type 2 diabetes [1]. Hyperglycaemia is measured using haemoglobin A1c (HbA1c) test which assesses the average level of blood glucose in the preceding 60–120 days. For diabetes patients an HbA1c target of 6.5% (48 mmol/mol) is recommended [2], [3] on the basis that lowers the risk of developing diabetic complications (i.e. kidney disease, heart disease). The UK Prospective Diabetes Study (UKPDS) established that intensive control of blood glucose in type 2 diabetes reduced the risk of microvascular complications, especially diabetic retinopathy, in patients with type 2 diabetes [4]. While the UKPDS did not find any effect of intensive blood glucose lowering on cardiovascular events, these were also found to be reduced during post-trial follow-up [5]. A systematic review of five trials confirmed that cardiovascular events were reduced through intensive control [6]. However, hypoglycaemia is a recognized hazard of intensive therapy, being more frequent in intensively treated patients [7]. Recent evidence suggests that hypoglycaemia may also be associated with adverse vascular events and all-cause mortality [7], [8]. The ACCORD study [9] found that intensive lowering of blood glucose (HbA1c target of 6.0%) was not associated with reduced cardiovascular events, but there was an increase in all-cause mortality in type 2 diabetic subjects at high risk of cardiovascular disease (CVD). A subsequent analysis of data from the ADVANCE study [10] suggested that there might be a threshold for the beneficial effect of glucose lowering with no benefit observed from reducing HbA1c below 7% for macrovascular events and mortality or below 6.5% for microvascular events [11].

Recently, Currie et al. [12] reported that either very high or very low HbA1c increased the risk of all-cause mortality in a large cohort of patients routinely treated in UK primary care. Their primary analysis did not allow for changes in HbA1c over time and instead used the mean of all HbA1c values subsequent to the index date. Although further time dependent analysis were carried out using yearly mean HbA1c, missing data was dealt with using last observation carried forward which can result in bias. This is a potentially important limitation, as Currie et al. [12] did not report on the completeness of HbA1c records and these may not have been routinely recorded during the period they examined (1986 to 2008). Riveline et al. [13] also noted that Currie et al.’s population may have had substantial heterogeneity since the UKPDS trial has led to significant changes in the management of Type 2 diabetes (i.e. risk modeling, health economics). In addition, existing treatment targets for HbA1C set by the NICE (HbA1C<7%) (2008) or the American Diabetes Association (HbA1C<7%) (2008) are not age specific and Pani et al. [14] underlined the importance of establishing whether age-specific treatment criteria would be appropriate.

This study aimed to evaluate short-term associations between HbA1c values recorded in clinical practice in primary care and subsequent risk of mortality in a post-UKPDS population. A case control study was implemented to establish an explicit temporal link between HbA1c values recorded in the previous 365 days and mortality risk, rather than utilizing HbA1c records that might cover a considerable length of time, as has been done previously [12]. We aimed to determine whether the risk of mortality was higher when the most recent HbA1c value in the preceding 365 days was either <6.5% or >9.0% compared to HbA1c values that were between 6.5% and 9%. Considering the scarce evidence for a possible age-depended influence of HbA1C levels on mortality [14], an additional aim of the present study was to explore potential age-associated differences in mortality rates for both low and high HbA1C levels.

## Methods

A nested case-control study was implemented using data from family practices contributing to the Clinical Practice Research Datalink (CPRD, formerly known as the General Practice Research Database) between 1 July 2000 and 30 April 2008. The CPRD contains comprehensive information on patients’ medical diagnoses, drug prescriptions, lifestyle advice, specialist referrals, laboratory tests, hospital admissions, and clinical findings (i.e. BMI, smoking, and blood pressure). For entry into the GPRD, practice data must be up to standard (UTS) for research as set out by the GPRD group. The validity of CPRD data for diagnoses and prescribing has been documented in several studies [15], [16]. Data for the present study was based on a research project developed in 2009 and thus the latest available data for analysis was to the end of December, 2008.

The case-control study was nested in a cohort of people with type 2 diabetes. A case-control design was preferred because it is more efficient than a cohort design for a rare outcome such as mortality. The study also intended to validate Currie et al.’s [12] findings by using a different approach to design. Participants were included in the cohort if they had ever been diagnosed with diabetes mellitus, or prescribed oral hypoglycemic drugs or insulin. Date of diabetes onset was defined as the earlier of first recorded medical or referral code for diabetes or first date of prescription of oral hypoglycemic drugs or insulin. Participants were excluded if they had ever been diagnosed with type 1 diabetes mellitus; were aged less than 30 years at diabetes onset; or were prescribed insulin within 180 days of diabetes onset. Participant follow-up started from the later of: date of onset of diabetes, date of registration with a CPRD practice, date at which the practice began contributing UTS data to CPRD, or 1 July 2000. Participants were censored when they transferred out of a CPRD practice, at the last date at which their practice contributed up to standard (UTS) data to CPRD, or on 30 April 2008.

Cases were participants who died while on follow-up in the cohort. For each eligible case, one control was randomly selected from the study cohort matched by gender, age category (<35, 35 to 44, 45 to 54, 55 to 64, 65 to 74, 75 to 84, 85+ years), and time since cohort entry (<90; 90 to 179; 180 to 364, 365+ days). Additional matching variables were considered unnecessary and might have resulted in overmatching. In addition, the study was sufficiently large to allow regression adjustment for multiple confounding variables [17]. One control per case was preferred as with large sample sizes as in the present study there is little gain in statistical power by including more than one control per case [18]. Each control was assigned the index date of their matched case.

### Measures

To evaluate both recent HbA1c and recent change in HbA1c the latest two values in the 365 days preceding the index date were identified. HbA1c values below 2.5% or over 25% were discarded as these values were considered implausible. The most recent HbA1c test results in the 365 days before the index date was used as the primary exposure and was classified as ‘low’ (HbA1c less than 6.5%), ‘normal’ (HbA1c between 6.5% and 9.0% mol/mol) or ‘high’ (HbA1c greater than 9.0%). A value of 6.5% is a commonly employed cut-off point in studies exploring HbA1c levels and mortality association [19], [20]. For instance, Zoungas et al. [20] suggested the 6.5% as the threshold above which there is an increase risk in microvascular events and death in diabetes patients. The same cut-point has also been recommended as a target for Type diabetes [2], [3]. The 9% cut-off point has been suggested to represent an indicator for ineffective blood glucose management in type 2 diabetes [3], [21]. Change in HbA1c represented the difference between the most recent value and the preceding HbA1c value if this was also recorded within 365 days of the index date. Change was classified as a decrease in HbA1c (decline in HbA1c of greater than −1%), no or marginal change (change between −1% and 1%), or increase in HbA1c (increase in HbA1c of greater than 1%).

To adjust for potential confounders of the relationship between HbA1c and mortality we identified diagnoses in the last 365 days of: coronary heart disease, arrhythmia, heart failure, stroke or transient ischemic attack, cancer, hypertension, renal failure, liver disease and malnutrition or malabsorption. Analysis also adjusted for treatment with lipid lowering therapies, including statins, within the last 365 days, most recent smoking status (3 categories: non-smoker, ex-smoker, current smoker) and BMI value recorded within the last 365 days (3 categories: normal/underweight, overweight, obese), and treatment with glucose lowering medications within 180 days (insulins, sulphonylureas, biguanides, pioglitazone, rosiglitazone, and other hypoglycemic medications). The 365 days time frame was informed by the likelihood that severe chronic illnesses will be monitored on at least yearly basis and thus using a 365 days period would allow identification of all patients previously diagnosed with a severe chronic condition. The use of 180 days period for drug therapy was based on the typical length of prescriptions in CPRD. The aim was to capture information concerning glucose therapy at the time of death. Participants who were not prescribed glucose lowering drugs were assumed to be on therapy with diet or exercise, though these interventions are not comprehensively recorded in GPRD.

### Statistical Analysis

Data were analyzed using conditional logistic regression in Stata MP version 11.2 (Stata corporation, College Station, Texas, USA) to estimate the association of mortality with low and high HbA1c levels using normal HbA1c level as the reference category. The initial model included adjustment only through matching (gender, age, time since cohort entry). The final model adjusted for all confounders listed above. The confounders were entered into the model as categorical explanatory variables. In order to evaluate effect modification, analyses were also carried out stratified by age group (age at index date: <55, 55–64, 65–74, 75–84, 85+ years). As with the primary analysis, conditional logistic regression models were fitted with and without adjustment for possible confounders of the relationship between HbA1c and mortality.

### Missing Data

Initial models used complete case analysis and included only matched sets where both the case and control had a valid HbA1c test result within the 365 days prior to the index date. For models examining change in HbA1c values, the complete case analysis included only matched sets where both the case and control had two valid HbA1c test results within the 365 days prior to the index date.

To evaluate the impact of missing data, multiple imputation was used to replace any missing values for the most recent two HbA1c tests. Multiple imputation was used to replace missing values for smoking status and BMI for patients without a record of these data in the previous 365 days. Multiple imputation was preferred because it is superior to other missing data approaches (i.e. mean replacement, last observation) even in situations where a large proportion of the data is missing [22]. Also, removing patients with missing data from the study (i.e. listwise) would result in a significant loss of the study sample, raising concerns about the validity of the results [23]. Data were imputed using multiple imputation by chained equations, which allows an appropriate imputation model to be defined for each variable. The “mi impute chained” command in Stata was used to implement predictive mean matching for HbA1c tests, and multinomial logistic regression for smoking and BMI category. Ten imputed datasets were generated. Predictive mean matching replaces each missing value by the observed value with the closest match on predicted value from the imputation regression model. Predictive mean matching was used as it is considered more robust to violation of the normality assumption of the regression model underlying the multiple imputation procedure and ensures that imputed values will be within the range of observed values [24]. Multinomial logistic regression was selected for imputing missing values for smoking and BMI category because these were constructed as a multinomial categorical variable (e.g. never smoked, ex-smoker, current smoker).

## Results

The cohort of participants included 97,450 participants from 226 family practices, who were followed up for a total of 386,738 person years (median 3.7 years per participant). All 16,585 cases that died during follow-up were matched to a suitable control and were included in the analyses. Participant characteristics for cases and controls are presented in Table 1. Cases tended to have marginally longer duration of diabetes relative to controls. Also a greater proportion of cases than controls were treated with insulin or sulphonylureas and a lower proportion were treated with biguanides, pioglitazone or rosiglitazone. A greater proportion of cases than controls had diagnosis in the prior 365 days of coronary heart disease, heart failure, stroke or transient ischemic attack, cancer, malnutrition or malabsorption, renal failure and liver disease. A smaller proportion of cases than controls had diagnosis of hypertension or treatment with lipid lowering medications. In addition, cases were more likely to be recorded as smokers and weigh less than controls.

**Table 1:** Participant characteristics for cases and controls.

Variable	Controls (n = 16585)	Cases (n = 16585)
**Male**	8569 (51.7)	8569 (51.7)
**Age at index date, years**		
<45	79 (0.5)	79 (0.5)
45 to 54	353 (2.1)	353 (2.1)
55 to 64	1378 (8.3)	1378 (8.3)
65 to 74	3842 (23.2)	3842 (23.2)
75 to 85	6496 (39.2)	6496 (39.2)
85+	4437 (26.8)	4437 (26.8)
**Duration diabetes (years)** a	5.5 (2.25, 10.63)	6.3 (2.55, 11.99)
**Duration of follow-up (years)** a	2.4 (1.00, 4.33)	2.5 (1.00, 4.44)
**Year of death**		
2000	847 (5.1)	847 (5.1)
2001	1858 (11.2)	1858 (11.2)
2002	2057 (12.4)	2057 (12.4)
2003	2154 (13.0)	2154 (13.0)
2004	2184 (13.2)	2184 (13.2)
2005	2315 (14.0)	2315 (14.0)
2006	2447 (14.8)	2447 (14.8)
2007	2478 (14.9)	2478 (14.9)
2008	245 (1.5)	245 (1.5)
**Smoking status**		
Non-smoker	7348 (44.3)	6312 (38.1)
Ex-smoker	6795 (41.0)	6451 (38.9)
Current-smoker	1657 (10.0)	2382 (14.4)
Missing	785 (4.7)	1440 (8.7)
**BMI category**		
Normal/underweight (BMI <25)	4297 (25.9)	5218 (31.5)
Overweight (25≤BMI <30)	6124 (36.9)	4736 (28.6)
Obese (BMI≥30)	4802 (29.0)	3771 (22.7)
Missing	1362 (8.2)	2860 (17.2)
**Glucose-lowering therapy in 180 days before index date:**		
Insulins	1328 (8.0)	2077 (12.5)
Sulphonylureas	6619 (39.9)	7254 (43.7)
Biguanides	6484 (39.1)	5531 (33.3)
Pioglitazone	270 (1.6)	167 (1.0)
Rosiglitazone	694 (4.2)	544 (3.3)
Other glucose lowering medications	253 (1.5)	234 (1.4)
Dietary advice only^b^	946 (5.7)	797 (4.8)
**Diagnoses & treatments 365 days before index date**		
Coronary heart disease	1099 (6.6)	2799 (16.9)
Arrhythmia	258 (1.6)	322 (1.9)
Heart failure	469 (2.8)	2176 (13.1)
Stroke or transient ischemic attack	350 (2.1)	1410 (8.5)
Hypertension	2820 (17.0)	1802 (10.9)
Cancer	818 (4.9)	3610 (21.8)
Malnutrition or malabsorption	97 (0.6)	204 (1.2)
Renal failure	297 (1.8)	1177 (7.1)
Liver disease	32 (0.2)	345 (2.1)
Treatment with lipid lowering medications	8064 (48.6)	6448 (38.9)

Values are frequency (percent) unless otherwise stated.

a figures are medians (interquartile range).

b No glucose lowering drugs were prescribed for these diabetes patients and most these patients were possible referred to dietary and exercise support.

At least one valid value of HbA1c was recorded in the 365 days before the index date for 79.9% of controls and 67.5% of cases (Table 2). Change in the last 365 days could be calculated for the 45.1% of controls and 33.0% of cases. Although mean HbA1c was higher for cases than controls, the average change in HbA1c was similar (0.14).

**Table 2:** Mean values and change in HbA1c in the last 365 days prior to the index date in cases and controls.

	Controls	Cases
	(n = 16585)	(n = 16585)
**Most recent HbA1c test in the last 365 days**		
Mean (standard deviation)	7.21 (1.38)	7.32 (1.66)
<6.5% (48 mmol/mol)	4018 (24.2)	3677 (22.2)
6.5% to 9.0% (48 to 75 mmol/mol)	7962 (48.0)	6040 (36.4)
>9.0% (75 mmol/mol)	1272 (7.7)	1485 (9.0)
Missing	3333 (20.1)	5383 (32.5)
**Change in HbA1c between two most recent tests in last 365 days**		
Mean change (standard deviation)	0.14 (0.97)	0.14 (1.20)
Decrease (decline of more than −1%)	439 (2.6)	517 (3.1)
No change (between −1% and 1%)	6231 (37.6)	4171 (25.2)
Increase (increase of greater than 1%)	818 (4.9)	779 (4.7)
Missing	9097 (54.9)	11118 (67.0)

Values are number (percent) unless otherwise stated.

The complete case analysis revealed that higher HbA1c (>9% or 75 mmol/mol) values were associated with increased odds (OR 1.58, CI: 1.37,1.82) of all-cause mortality. Low HbA1c (<6.5%) values were also associated with increased odds of all-cause mortality (OR = 1.22, CI: 1.11,1.34) in comparison to normal HbA1c levels after adjustment for study confounders (Table 3). Since not all cases and controls had a valid HbA1c test result in the last 365 days, only 9,241 of the total of 16,585 pairs of matched case and controls were available for the complete case analysis. Participants with missing HbA1c values were more likely to be cases and were also younger, less intensively treated with lipid lowering medications and diabetes medications, less likely to have diagnosis of CHD, heart failure, renal disease or hypertension and had shorter length of follow-up time at their index date. Therefore, it appears unlikely that data were missing completely at random and so it is possible that bias may be present in the complete case analysis. Multiple imputation analyses results also suggested that both low and high HbA1c levels were associated with increased risk of mortality in comparison to normal HbA1c levels (Table 3), but the effect sizes were somewhat smaller relative to the complete case analysis.

**Table 3:** Association between mortality and HbA1c.

Model	Complete case		Analysis	Multiple			Imputation
	Matched OR		Adjusted OR^a^	Matched OR			Adjusted OR^a^
	(95% CI)		(95% CI)	(95% CI)			(95% CI)
**Model 1: Most recent HbA1c**							
Number of matched pairs	9241	7902			16585		16585
<6.5%^b^	1.21 (1.14, 1.29)	1.22 (1.11, 1.34)			1.14 (1.08, 1.20)		1.12 (1.04, 1.20)
>9.0%^b^	1.62 (1.46, 1.78)	1.58 (1.37, 1.82)			1.40 (1.29, 1.53)		1.29 (1.16, 1.44)
**Model 2: Most recent value and change in HbA1c**							
Number of matched pairs	2739	2481			16585	16585	
<6.5%^b^	1.24 (1.10, 1.41)	1.28 (1.08, 1.51)			1.16 (1.10, 1.22)		1.13 (1.05, 1.21)
>9.0%^b^	1.52 (1.25, 1.85)	1.46 (1.11, 1.91)			1.31 (1.19, 1.43)		1.23 (1.09, 1.37)
Decrease^c^	1.55 (1.24, 1.93)	1.50 (1.11, 2.02)			1.28 (1.13, 1.45)		1.21 (1.01, 1.45)
Increase^c^	1.58 (1.33, 1.87)	1.39 (1.10, 1.75)			1.25 (1.14, 1.38)		1.16 (1.03, 1.30)

Figures are odds ratios (OR), 95% confidence intervals (CI).

a adjusted for comorbidity, lipid lowering medication, smoking, BMI and diabetes drug utilisation.

b 6.5% to 9.0% as reference category.

c ‘no change’ as reference category.

From the complete case model and multiple imputation model, changes in HbA1c within the last 365 days also appeared to be associated with increased mortality risk. Adjusting for study confounders, decreasing HbA1c levels prior to death were associated with 1.50 (CI: 1.11, 2.02) greater odds of all-cause mortality compared to no HbA1c levels change. A lower and effect size was observed with respect to increasing HbA1c levels (OR = 1.39, CI: 1.10,1.75). Fully specified models are detailed in the Supplementary material (Table S1 in File S1).

When the association between HbA1c and mortality was examined separately by age group, both the complete case analysis and multiple imputation models indicated that both low and high HbA1c was significantly associated with increased risk of mortality among participants aged 55 to 74 (Table 4). In addition, multiple imputation results indicated that high HbA1c (>9%) were significantly associated with increased risk of all-cause mortality (OR = 1.29, CI: 1.08,1.53) among the 75 to 84 age groups compared to normal HbA1c (6.5% to 9%). Both complete case analysis and multiple imputation models indicated that the odds ratio for low HbA1c (<6.5%) was greatest in participants aged less than 55 years old (2.05 (CI: 0.83,5.06) for complete case analysis and 1.53 (CI:0.84,2.79) for multiple imputation), and declined steadily with older age to become close to one for participants aged 85 and older (1.05 (CI:0.87,1.26) for complete case analysis and 1.04 (CI:0.92,1.17) for multiple imputation). A similar declining trend with age was observed with respect to high HbA1c levels (apart from the youngest age group). Fully specified models are detailed in the Supplementary material (Table S2 in File S1).

**Table 4:** Association between mortality and HbA1c, stratified by age group.

Model	Age group (years)				
	<55	55–64	65–74	75–84	85+
**COMPLETE CASE**					
**Matched OR**					
**Number of matched pairs**	179	793	2353	3818	2098
<6.5%^a^	2.16 (1.31, 3.56)	1.51 (1.20, 1.91)	1.31 (1.15, 1.50)	1.19 (1.08, 1.31)	1.03 (0.91, 1.18)
>9.0%^a^	1.59 (0.91, 2.76)	1.65 (1.26, 2.15)	1.93 (1.59, 2.33)	1.62 (1.37, 1.91)	1.28 (1.02, 1.62)
**Adjusted OR** b					
**Number of matched pairs**	164	737	2149	3307	1545
<6.5% a	2.05 (0.83, 5.06)	1.67 (1.14,2.46)	1.37 (1.12, 1.67)	1.19 (1.03, 1.37)	1.05 (0.87, 1.26)
>9.0%^a^	1.72 (0.76, 3.90)	1.84 (1.19, 2.84)	1.89 (1.43, 2.50)	1.60 (1.27, 2.02)	1.28 (0.92, 1.77)
**MULTIPLE IMPUTATION**					
**Number of matched pairs**	432	1378	3842	6496	4437
**Matched OR**					
<6.5%^a^	1.70 (1.19, 2.43)	1.32 (1.09, 1.60)	1.16 (1.04, 1.30)	1.11 (1.03, 1.21)	1.08 (0.98, 1.19)
>9.0%^a^	1.34 (0.89, 2.00)	1.49 (1.18, 1.87)	1.60 (1.37, 1.87)	1.41 (1.23, 1.62)	1.20 (1.01, 1.42)
**Adjusted OR** b					
<6.5%^a^	1.53 (0.84, 2.79)	1.42 (1.04, 1.92)	1.19 (1.01, 1.41)	1.08 (0.96, 1.21)	1.04 (0.92, 1.17)
>9.0%^a^	1.20 (0.70, 2.07)	1.64 (1.13, 2.38)	1.46 (1.19, 1.80)	1.29 (1.08, 1.53)	1.15 (0.94, 1.41)

Figures are odds ratios (95% confidence intervals).

a 6.5% to 9.0% as reference category;

b adjusted for comorbidity, lipid lowering medication, smoking, BMI and diabetes drug utilisation.

## Discussion

In a population-based study it was revealed that both low and high HbA1c values are associated with increased short-term risk of all-cause mortality. In adults diagnosed with diabetes in primary care there was a 60% increase in the odds of all-cause mortality associated with high HbA1c levels and a 40% increase in the odds of all-cause mortality associated with low HbA1c levels. Employing a post-UKPDS population, the study also demonstrates that both increases and decreases in HbA1c values prior to death are associated with increased risk of mortality.

A possible age-associated effect for the relationship between HbA1c and mortality risk was observed. In particular, the strength of the association between HbA1c levels and all-cause mortality showed a consistent decline from younger age group (<55 years of age) to the older age group (>85 years of age) suggesting a possible age-associated impact of HbA1c on all-cause mortality. Interestingly, for older people (75 to 84 years old) only high HbA1c values were significantly associated with increased risk of mortality. Current treatment recommendations for type 2 diabetes do not take into account patients’ age and the findings of the present study suggest that younger and older diabetes patients may benefit from different HbA1c treatment targets.

Although the mortality risks were elevated at all ages, the results are presented by age because of the clinical importance. Higher relative mortality at younger ages may be expected because deaths from other causes are less frequent, while in older age high death rates from other background causes are expected.

The finding that high HbA1c levels are associated with increased risk of mortality could be partially explained by the fact that high HbA1c levels are associated with increased risk of diabetic macrovascular and microvascular complications [25], [26]. These may contribute to deteriorating kidney function and anemia have also been suggested to account for increased risk of mortality associated with increased levels of HbA1c [14]. The association of low HbA1c with mortality might be explained through hypoglycaemia [27] or through association with liver disease [28]. Low HbA1c values may sometimes indicate the presence of morbidity and worse health.

Currie et al. [12] found that both high and low HbA1c levels may increase the risk of all-cause mortality. The present study substantiates the findings of Currie et al. [10] in a post-UKPDS population and extends them by documenting a possible age-associated relationship of HbA1c levels with the risk of all-cause mortality. Currie et al. [12] study includes older patients (aged 50 years and above) and thus it was not possible to explore whether the observed association between HbA1c and mortality in older patients extends to younger diabetes patients. Our study findings imply that intensive HbA1c lowering therapy increases mortality risk across the age continuum, but also that the greater relative risk was observed in younger (<55 years of age) diabetes patients. The findings of the present study also appear to be consistent with those found in the ACCORD and VADT trials [29], [30]. In an earlier study, Gerstein et al. [25] suggested that stringent HbA1c levels might cause an excess risk of all-cause mortality. By contrast, Ray et al. [31] in a recent meta-analysis of five RCTs concluded that a decrease in HbA1c levels were not associated with reducing risk of all-cause mortality. More recently, Boussageon et al. [32] and Hemmingsen et al. [33] reached a similar conclusion in two meta-analyses of RCTs. The present study raises questions concerning the application of trial evidence to a wider primary care. By addressing some of the previous studies methodological problems, the present study helps clarify the current debate and established that both high and low HbA1c level can be associated with an increased risk for all-cause mortality.

This study has notable strengths including the consideration of different HbA1c levels and short-term changes in HbA1c values with use of multiple imputation to explore potential bias from missing values, to provide evidence about the impact of HbA1c on mortality in a nationally representative cohort of diabetic patients. However, several limitations are worth mentioning. The study did not include information on patients’ exercise or dietary habits which may modify medication effects and might partially explain associations with mortality. We did not explore whether stable or changing HbA1c levels are more strongly associated with mortality. It is possible that the association of low HbA1c levels with mortality could mask rather unstable HbA1c levels over time. The findings that both increased and decreased HbA1c levels were associated with higher mortality likelihood point towards such possibility. Causes of death were not available for analysis limiting our ability to explore whether disorders of blood glucose were immediately related to patient death or whether other conditions including comorbid disorders or fatal accidents may be responsible for greater mortality rates in this population. Adjusting for comorbid disorders ensured however that comorbidity might not be the sole explanations for the findings.

The small proportion of patients who experienced a decrease or increase in HbA1c values between the last two measurements limited our ability to conduct more detailed analyses such the effect of decrease or increase in HbA1c values within the low (<6.5%) and high (>9.0%) categories. The study cannot rule out the possibility of residual confounding which may explain the observed association between Hb1Ac and mortality. Additionally, because of the inconsistent recording of dietary data in CPRD it was not possible to compare the reduction in HbA1c levels due to diet or medication. However, most patients on diabetes medication will also be offered dietary advice, making difficult any definite distinction about differential reduction in HbA1c levels between diet and medication in observational studies.

It is likely that mortality risks may differ for different drugs and drug combinations and several studies [34], [35] have linked sulphonylurea drugs with increased mortality and glitazone therapy with increased risk of cardiovascular events [36], [37]. These are important hypotheses that deserve to be evaluated as primary hypotheses of interest in purposely designed studies, rather than as secondary analyses in studies implemented for other purposes such as the present study.

### Conclusion

The present findings suggest that an HbA1c target of less than 6.5% or 48 mmol/mol might be too low for some patients and large reductions, or increases, in HbA1c levels should be approached cautiously. The findings also point to potential age-related differences in HbA1c levels and mortality rates with younger diabetes patients being at relatively greater risk of mortality associated with low HbA1c levels even though absolute risks are smaller at young ages. However, this finding needs replication in future studies before making any definitive recommendations regarding the development of different HbA1c treatment targets for younger and older age diabetes patients.

## Supporting Information

File S1 **Table S1.** Association between mortality and HbA1c. **Table S2.** Association between mortality and HbA1c, stratified by age group.
(PDF)
