# Supplementary material for: Recent HbA1c Values and Mortality Risk in Type 2 Diabetes. Population-Based Case-Control Study
Source: PLoS One. 2013 Jul 5;8(7):e68008. doi: 10.1371/journal.pone.0068008 (PMC3702542; doi:10.1371/journal.pone.0068008)
Supplement: File S1 — Table S1. Association between mortality and HbA1c. Table S2. Association between mortality and HbA1c, stratified by age group. (PDF) [file pone.0068008.s001.pdf]

Table 1: Association between mortality and HbA1c

COMPLETE CASE ANALYSIS

```
MODEL 1 (unadjusted): HbA1c <6.5% (coded 0), HbA1c>9.0% (coded 2) versus base HbA1c >=6.5% & <=9.0% (coded 1)
clogit _c ib1.range_hba1c365, group(_set)
note: 5972 groups (5972 obs) dropped because of all positive or all negative outcomes.

Iteration 0:   log likelihood = -6352.0411
Iteration 1:   log likelihood = -6352.0065
Iteration 2:   log likelihood = -6352.0065

Conditional (fixed-effects) logistic regression   Number of obs   =      18482
                                                  LR chi2(2)      =     106.73
                                                  Prob > chi2     =     0.0000
Log likelihood = -6352.0065                       Pseudo R2       =     0.0083

------------------------------------------------------------------------------
        _case |      Coef.   Std. Err.      z    P>|z|     [95% Conf. Interval]
-------------+----------------------------------------------------------------
range_hba1c365 |
           0  |   .1930619   .0328261     5.88   0.000     .1287239    .2573999
           2  |   .4801367   .0505916     9.49   0.000      .380979    .5792944
------------------------------------------------------------------------------
```

Table 1: Association between mortality and HbA1c

COMPLETE CASE ANALYSIS

MODEL 1 (adjusted): HbA1c <6.5% (coded 0), HbA1c>9.0% (coded 2) versus base HbA1c >=6.5% & <=9.0% (coded 1)

```
. #delimit ;

delimiter now ;

. clogit _c ib1.range_hba1c365
>         i.smoking
>         i.range_bmi_365
>         duration_diab
>         insulins sulphonylureas biguanides other_diabetes pioglitazone rosiglitazone
>         CHD_365 arrhythmia_365 HF_365 renal_365 hypertension_365 stroke_365 neoplasms_365 mal_365 liver_365
>         lipid_d_365
>         , group(_set)
> ;

note: 6556 groups (6556 obs) dropped because of all positive or all negative outcomes.

Iteration 0:   log likelihood = -3683.5797
Iteration 1:   log likelihood = -3674.9492
Iteration 2:   log likelihood = -3674.9328
Iteration 3:   log likelihood = -3674.9328


Conditional (fixed-effects) logistic regression   Number of obs   =      15804
                                                  LR chi2(23)     =    3604.63
                                                  Prob > chi2     =     0.0000
Log likelihood = -3674.9328                       Pseudo R2       =     0.3291
```

```
------------------------------------------------------------------------------------
               _case |      Coef.   Std. Err.      z    P>|z|     [95% Conf. Interval]
---------------------+--------------------------------------------------------------
      range_hba1c365 |
                  0  |   .2005416   .0473605     4.23   0.000     .1077167    .2933664
                  2  |   .4575419    .071567     6.39   0.000     .3172732    .5978105
                     |
             smoking |
                  1  |   .2314273   .0462788     5.00   0.000     .1407225    .3221321
                  2  |   .5600431    .067738     8.27   0.000     .4272791    .6928071
                     |
range_bmi_365_simple |
                  2  |  -.4937741   .0503051    -9.82   0.000    -.5923704   -.3951778
                  3  |  -.4133784   .0548605    -7.54   0.000    -.5209031   -.3058537
                     |
       duration_diab |   .0183428   .0033445     5.48   0.000     .0117878    .0248979
            insulins |   .3300296   .0743057     4.44   0.000     .1843931    .4756662
      sulphonylureas |   .1371034   .0442751     3.10   0.002     .0503257    .2238811
          biguanides |  -.0520454   .0431143    -1.21   0.227     -.136548    .0324571
      other_diabetes |  -.2453124   .1534231    -1.60   0.110    -.5460161    .0553912
        pioglitazone |  -.2092597   .1482464    -1.41   0.158    -.4998172    .0812979
       rosiglitazone |  -.1602362   .0915184    -1.75   0.080    -.3396089    .0191366
             CHD_365 |   1.318451   .0706081    18.67   0.000     1.180062     1.45684
      arrhythmia_365 |  -.1333878   .1499036    -0.89   0.374    -.4271935    .1604179
              HF_365 |    1.75869   .0987242    17.81   0.000     1.565194    1.952186
           renal_365 |   1.319158   .1137143    11.60   0.000     1.096282    1.542034
    hypertension_365 |  -.4791182    .058055    -8.25   0.000    -.5929039   -.3653325
          stroke_365 |   1.751312   .1147237    15.27   0.000     1.526458    1.976167
       neoplasms_365 |       1.97   .0732701    26.89   0.000     1.826393    2.113607
             mal_365 |    .465199    .216069     2.15   0.031     .0417115    .8886865
           liver_365 |   1.999208   .2909514     6.87   0.000     1.428953    2.569462
         lipid_d_365 |  -.5421524   .0459839   -11.79   0.000    -.6322791   -.4520257
------------------------------------------------------------------------------------

. #delimit cr
delimiter now cr
```

Table 1: Association between mortality and HbA1c

COMPLETE CASE ANALYSIS

```
MODEL 2 (unadjusted):
  HbA1c <6.5% (coded 0), HbA1c>9.0% (coded 2) versus base HbA1c >=6.5% & <=9.0% (coded 1)
  Change HbA1c <1% (coded 0),  change HbA1c>1.0% (coded 2) versus base change HbA1c >=1% & <=1% (coded 1)

. #delimit ;
delimiter now ;
. clogit _c
>         ib1.range_hba1c365
>         ib1.change_cat
>         , group(_set)
>         ;
note: 7477 groups (7477 obs) dropped because of all positive or all negative outcomes.

Iteration 0:   log likelihood = -1859.2073
Iteration 1:   log likelihood = -1859.1174
Iteration 2:   log likelihood = -1859.1174

Conditional (fixed-effects) logistic regression   Number of obs   =       5478
                                                  LR chi2(4)      =      78.83
                                                  Prob > chi2     =     0.0000
Log likelihood = -1859.1174                       Pseudo R2       =     0.0208

--------------------------------------------------------------------------------
         _case |      Coef.   Std. Err.      z    P>|z|     [95% Conf. Interval]
---------------+----------------------------------------------------------------
range_hba1c365 |
            0  |   .2187613   .0627596     3.49   0.000     .0957548    .3417678
            2  |   .4198971   .1009382     4.16   0.000     .2220618    .6177324
               |
 change_cat365 |
            0  |   .4372099   .1131133     3.87   0.000     .2155118     .658908
            2  |   .4562241   .0871577     5.23   0.000     .2853981    .6270501
--------------------------------------------------------------------------------
```

Table 1: Association between mortality and HbA1c

COMPLETE CASE ANALYSIS

MODEL 2 (adjusted):
HbA1c <6.5% (coded 0), HbA1c>9.0% (coded 2) versus base HbA1c >=6.5% & <=9.0% (coded 1)
Change HbA1c <1% (coded 0), change HbA1c>1.0% (coded 2) versus base change HbA1c >=1% & <=1% (coded 1)

```
. #delimit cr
delimiter now cr
.
.
. #delimit ;
delimiter now ;
. clogit _c
>         ib1.range_hba1c365
>         ib1.change_cat
>         i.smoking
>         i.range_bmi_365_simple
>         duration_diab
>         insulins sulphonylureas biguanides other_diabetes pioglitazone rosiglitazone
>         CHD_365 arrhythmia_365 HF_365 renal_365 hypertension_365 stroke_365 neoplasms_365 mal_365 liver_365
>     lipid_d_365
>         , group(_set)
> ;
note: 7223 groups (7223 obs) dropped because of all positive or all negative outcomes.

Iteration 0:   log likelihood = -1154.0068
Iteration 1:   log likelihood = -1149.7373
Iteration 2:   log likelihood = -1149.7293
Iteration 3:   log likelihood = -1149.7293

Conditional (fixed-effects) logistic regression   Number of obs   =       4962
                                                  LR chi2(25)     =    1139.94
                                                  Prob > chi2     =     0.0000
Log likelihood = -1149.7293                       Pseudo R2       =     0.3314
```

_case	Coef.	Std. Err.	z	P>|z|	[95% Conf.	Interval]
range_hba1c365						
0	.2445017	.0869312	2.81	0.005	.0741197	.4148838
2	.3770757	.1377107	2.74	0.006	.1071677	.6469837
change_cat365						
0	.4042356	.1535666	2.63	0.008	.1032505	.7052207
2	.3265555	.1180714	2.77	0.006	.0951398	.5579712
smoking						
1	.1807346	.0838529	2.16	0.031	.016386	.3450832
2	.6074898	.1258009	4.83	0.000	.3609246	.854055
range_bmi_365_simple						
2	-.4473001	.0914452	-4.89	0.000	-.6265294	-.2680708
3	-.2476695	.0993096	-2.49	0.013	-.4423128	-.0530263
duration_diab	.0170575	.0058433	2.92	0.004	.0056048	.0285101
insulins	.3210693	.1284085	2.50	0.012	.0693932	.5727453
sulphonylureas	.1353912	.0798747	1.70	0.090	-.0211603	.2919427
biguanides	-.0533053	.0779162	-0.68	0.494	-.2060183	.0994077
other_diabetes	.1273544	.2625282	0.49	0.628	-.3871915	.6419003
pioglitazone	-.1357892	.2438041	-0.56	0.578	-.6136364	.342058
rosiglitazone	-.0259665	.1408634	-0.18	0.854	-.3020537	.2501207
CHD_365	1.379179	.11939	11.55	0.000	1.145179	1.61318
arrhythmia_365	-.3555733	.2482589	-1.43	0.152	-.8421518	.1310052
HF_365	1.463429	.1663275	8.80	0.000	1.137433	1.789425
renal_365	1.320239	.1875793	7.04	0.000	.9525899	1.687887
hypertension_365	-.3971462	.100581	-3.95	0.000	-.5942814	-.200011
stroke_365	1.71362	.2034824	8.42	0.000	1.314802	2.112438
neoplasms_365	1.978014	.1320039	14.98	0.000	1.719291	2.236737
mal_365	.5842321	.3732155	1.57	0.117	-.1472568	1.315721
liver_365	1.766563	.4664999	3.79	0.000	.85224	2.680886
lipid_d_365	-.6524839	.0848677	-7.69	0.000	-.8188215	-.4861463

Table 2. Association between mortality and HbA1c, stratified by age group
COMPLETE CASE ANALYSIS

```
Unadjusted: HbA1c <6.5% (coded 0), HbA1c>9.0% (coded 2) versus base HbA1c >=6.5% & <=9.0% (coded 1)
note:
Agecat2
 45 "<55"
 55 "55-64"
 65 "65-74"
 75 "75-84"
 85 "85+"

. #delimit ;
delimiter now ;
. bysort agecat2: clogit _c
>         ib1.range_hba1c365
>         , group(_set)
> ;
```

```
-------------------------------------------------------------------------------------------------------------
```

-> agecat2 = 45

```
note: 195 groups (195 obs) dropped because of all positive or
      all negative outcomes.

Iteration 0:   log likelihood = -119.00784
Iteration 1:   log likelihood = -118.96832
Iteration 2:   log likelihood = -118.96832

Conditional (fixed-effects) logistic regression   Number of obs   =        358
                                                  LR chi2(2)      =      10.21
                                                  Prob > chi2     =     0.0061
Log likelihood = -118.96832                       Pseudo R2       =     0.0411
------------------------------------------------------------------------------
         _case |      Coef.   Std. Err.      z    P>|z|     [95% Conf. Interval]
---------------+--------------------------------------------------------------
range_hba1c365 |
            0  |   .7722753   .2545362     3.03   0.002     .2733934    1.271157
            2  |   .4615672   .2829138     1.63   0.103    -.0929337    1.016068
------------------------------------------------------------------------------
```

-> agecat2 = 55

```
note: 493 groups (493 obs) dropped because of all positive or all negative outcomes.

Iteration 0:   log likelihood = -539.31624
Iteration 1:   log likelihood = -539.25577
Iteration 2:   log likelihood = -539.25577

Conditional (fixed-effects) logistic regression   Number of obs   =       1586
                                                  LR chi2(2)      =      20.82
                                                  Prob > chi2     =     0.0000
Log likelihood = -539.25577                       Pseudo R2       =     0.0189

------------------------------------------------------------------------------
       _case |      Coef.   Std. Err.      z    P>|z|     [95% Conf. Interval]
-------------+----------------------------------------------------------------
range_hba1c365 |
          0  |    .413509   .1199183     3.45   0.001     .1784734    .6485446
          2  |   .4996181   .1354982     3.69   0.000     .2340465    .7651898
------------------------------------------------------------------------------
```

-> agecat2 = 65

```
note: 1253 groups (1253 obs) dropped because of all positive or all negative outcomes.

Iteration 0:   log likelihood = -1604.2135
Iteration 1:   log likelihood = -1604.1471
Iteration 2:   log likelihood = -1604.1471

Conditional (fixed-effects) logistic regression   Number of obs   =       4706
                                                  LR chi2(2)      =      53.66
                                                  Prob > chi2     =     0.0000
Log likelihood = -1604.1471                       Pseudo R2       =     0.0164
------------------------------------------------------------------------------
       _case |      Coef.   Std. Err.      z    P>|z|     [95% Conf. Interval]
-------------+----------------------------------------------------------------
range_hba1c365 |
          0  |   .2720831   .0678299     4.01   0.000     .1391389    .4050273
          2  |   .6557901   .0968334     6.77   0.000     .4660001    .8455801
------------------------------------------------------------------------------
```

-> agecat2 = 75

```
note: 2230 groups (2230 obs) dropped because of all positive or all negative outcomes.

Iteration 0:   log likelihood = -2626.8676
Iteration 1:   log likelihood = -2626.8631
Iteration 2:   log likelihood = -2626.8631

Conditional (fixed-effects) logistic regression   Number of obs   =       7636
                                                  LR chi2(2)      =      39.15
                                                  Prob > chi2     =     0.0000
Log likelihood = -2626.8631                       Pseudo R2       =     0.0074

------------------------------------------------------------------------------
       _case |      Coef.   Std. Err.      z    P>|z|     [95% Conf. Interval]
-------------+----------------------------------------------------------------
range_hba1c365 |
          0  |   .1748962   .0504366     3.47   0.001     .0760423    .2737501
          2  |    .480743   .0839639     5.73   0.000     .3161768    .6453092
------------------------------------------------------------------------------
```

-> agecat2 = 85

```
note: 1801 groups (1801 obs) dropped because of all positive or all negative outcomes.

Iteration 0:   log likelihood = -1451.9943
Iteration 1:   log likelihood = -1451.9839
Iteration 2:   log likelihood = -1451.9839

Conditional (fixed-effects) logistic regression   Number of obs   =       4196
                                                  LR chi2(2)      =       4.48
                                                  Prob > chi2     =     0.1066
Log likelihood = -1451.9839                       Pseudo R2       =     0.0015
------------------------------------------------------------------------------
       _case |      Coef.   Std. Err.      z    P>|z|     [95% Conf. Interval]
-------------+----------------------------------------------------------------
range_hba1c365 |
          0  |   .0337481   .0661471     0.51   0.610    -.0958978     .163394
          2  |   .2493193   .1182161     2.11   0.035       .01762    .4810185
------------------------------------------------------------------------------
```

Table 2. Association between mortality and HbA1c, stratified by age group

COMPLETE CASE ANALYSIS

Adjusted: HbA1c <6.5% (coded 0), HbA1c>9.0% (coded 2) versus base HbA1c >=6.5% & <=9.0% (coded 1)

note:

```
Agecat2
 45 "<55"
 55 "55-64"
 65 "65-74"
 75 "75-84"
 85 "85+"

. #delimit cr
delimiter now cr
.
. #delimit ;
delimiter now ;
. bysort agecat2: clogit _c
>         ib1.range_hba1c365
>         i.smoking
>         i.range_bmi_365_simple
>         duration_diab
>         insulins sulphonylureas biguanides other_diabetes pioglitazone rosiglitazone
>         CHD_365 arrhythmia_365 HF_365 renal_365 hypertension_365 stroke_365 neoplasms_365 mal_365 liver_365
>     lipid_d_365
>         if within365==1
>         , group(_set)
> ;



-------------------------------------------------------------------------------------------------------------------
```

-> agecat2 = 45

```
note: 187 groups (187 obs) dropped because of all positive or all negative outcomes.

Iteration 0:   log likelihood = -85.009567
Iteration 1:   log likelihood = -71.349086
Iteration 2:   log likelihood = -68.347602
Iteration 3:   log likelihood = -67.966509
Iteration 4:   log likelihood = -67.901226
Iteration 5:   log likelihood =  -67.88707
Iteration 6:   log likelihood = -67.883577
Iteration 7:   log likelihood = -67.882869
Iteration 8:   log likelihood = -67.882717
Iteration 9:   log likelihood = -67.882682
Iteration 10:  log likelihood = -67.882674
Iteration 11:  log likelihood = -67.882672

Conditional (fixed-effects) logistic regression   Number of obs   =        328
                                                  LR chi2(23)     =      91.59
                                                  Prob > chi2     =     0.0000
Log likelihood = -67.882672                       Pseudo R2       =     0.4028

------------------------------------------------------------------------------------
               _case |      Coef.   Std. Err.      z    P>|z|     [95% Conf. Interval]
---------------------+--------------------------------------------------------------
      range_hba1c365 |
                   0 |   .7185937   .4610218     1.56   0.119    -.1849924     1.62218
                   2 |   .5411209   .4183168     1.29   0.196     -.278765    1.361007
                     |
             smoking |
                   1 |  -.1952899   .4000683    -0.49   0.625    -.9794093    .5888295
                   2 |   .5996848   .3990024     1.50   0.133    -.1823454    1.381715
                     |
range_bmi_365_simple |
                   2 |   .1328787   .4808553     0.28   0.782    -.8095803    1.075338
                   3 |   .1450066   .4699407     0.31   0.758    -.7760603    1.066074
                     |
       duration_diab |  -.0186729   .0451909    -0.41   0.679    -.1072454    .0698997
```

```
         insulins |   .6817845   .5821464     1.17   0.242    -.4592015    1.822771
    sulphonylureas |  -.0503144   .3605645    -0.14   0.889    -.7570079    .6563792
        biguanides |  -.0804507   .3444447    -0.23   0.815      -.75555    .5946486
   other_diabetes |  -.4369955   1.239242    -0.35   0.724    -2.865866    1.991875
     pioglitazone |   .1450046   .9889509     0.15   0.883    -1.793303    2.083313
    rosiglitazone |  -1.258746   .7320824    -1.72   0.086    -2.693602    .1761088
          CHD_365 |   2.078284   .7339798     2.83   0.005     .6397103    3.516858
   arrhythmia_365 |   1.582979   1.759167     0.90   0.368    -1.864925    5.030883
           HF_365 |    20.0464   6439.991     0.00   0.998     -12602.1     12642.2
        renal_365 |   20.01892   8983.556     0.00   0.998    -17587.43    17627.46
 hypertension_365 |    -.21268   .4677645    -0.45   0.649    -1.129482    .7041217
       stroke_365 |   20.41648   11420.32     0.00   0.999    -22362.99    22403.83
    neoplasms_365 |   2.035634   .6724572     3.03   0.002      .717642    3.353626
          mal_365 |   17.67945   8265.754     0.00   0.998     -16182.9    16218.26
        liver_365 |   19.36784   4397.329     0.00   0.996    -8599.239    8637.975
      lipid_d_365 |  -.1777884   .3199444    -0.56   0.578    -.8048678     .449291
------------------------------------------------------------------------------------
```

-> agecat2 = 55

```
note: 523 groups (523 obs) dropped because of all positive or all negative outcomes.

Iteration 0:   log likelihood = -259.24386
Iteration 1:   log likelihood = -255.11067
Iteration 2:   log likelihood = -255.04508
Iteration 3:   log likelihood = -255.04497
Iteration 4:   log likelihood = -255.04497

Conditional (fixed-effects) logistic regression   Number of obs   =       1474
                                                  LR chi2(23)     =     511.61
                                                  Prob > chi2     =     0.0000
Log likelihood = -255.04497                       Pseudo R2       =     0.5007
```

_case	Coef.	Std. Err.	z	P>|z|	[95% Conf.	Interval]
range_hba1c365						
0	.5156754	.1956516	2.64	0.008	.1322053	.8991454
2	.6072374	.221882	2.74	0.006	.1723566	1.042118
smoking						
1	.5085423	.1844345	2.76	0.006	.1470573	.8700273
2	1.110507	.2319354	4.79	0.000	.6559224	1.565092
range_bmi_365_simple						
2	-.7614413	.2500184	-3.05	0.002	-1.251468	-.2714142
3	-.2529684	.2370078	-1.07	0.286	-.7174952	.2115585
duration_diab	.0471292	.0168469	2.80	0.005	.0141098	.0801486
insulins	.1131183	.2572719	0.44	0.660	-.3911253	.6173619
sulphonylureas	.3219639	.1823279	1.77	0.077	-.0353922	.6793199
biguanides	-.405332	.1708982	-2.37	0.018	-.7402863	-.0703777
other_diabetes	-.895775	.5288704	-1.69	0.090	-1.932342	.1407919
pioglitazone	.3671123	.4677496	0.78	0.433	-.5496599	1.283885
rosiglitazone	-.216202	.3484588	-0.62	0.535	-.8991686	.4667646
CHD_365	1.637323	.2704841	6.05	0.000	1.107184	2.167462
arrhythmia_365	.4665781	.6216273	0.75	0.453	-.751789	1.684945
HF_365	2.27899	.4955161	4.60	0.000	1.307796	3.250183
renal_365	2.653232	.6558214	4.05	0.000	1.367846	3.938619
hypertension_365	-.2511048	.2289406	-1.10	0.273	-.69982	.1976105
stroke_365	1.082578	.5202356	2.08	0.037	.0629354	2.102221
neoplasms_365	4.03799	.4711319	8.57	0.000	3.114589	4.961392
mal_365	2.179747	1.194515	1.82	0.068	-.1614597	4.520955
liver_365	2.22902	.638469	3.49	0.000	.9776438	3.480396
lipid_d_365	-.457141	.1818129	-2.51	0.012	-.8134877	-.1007943

-> agecat2 = 65

```
note: 1379 groups (1379 obs) dropped because of all positive or all negative outcomes.

Iteration 0:   log likelihood = -876.26866
Iteration 1:   log likelihood = -872.13429
Iteration 2:   log likelihood =  -872.1131
Iteration 3:   log likelihood =  -872.1131

Conditional (fixed-effects) logistic regression   Number of obs   =       4298
                                                  LR chi2(23)     =    1234.92
                                                  Prob > chi2     =     0.0000
Log likelihood =  -872.1131                       Pseudo R2       =     0.4145

------------------------------------------------------------------------------------
               _case |      Coef.   Std. Err.      z    P>|z|     [95% Conf. Interval]
---------------------+--------------------------------------------------------------
      range_hba1c365 |
                   0 |    .312518    .101265     3.09   0.002     .1140422    .5109939
                   2 |   .6358492   .1431199     4.44   0.000     .3553394    .9163591
                     |
             smoking |
                   1 |   .4322137    .096976     4.46   0.000     .2421442    .6222831
                   2 |   .5900068   .1269526     4.65   0.000     .3411843    .8388293
                     |
range_bmi_365_simple |
                   2 |  -.5008471   .1134467    -4.41   0.000    -.7231985   -.2784958
                   3 |  -.4740217   .1135734    -4.17   0.000    -.6966215   -.2514219
                     |
       duration_diab |   .0206855   .0075022     2.76   0.006     .0059814    .0353896
            insulins |   .6207265   .1481007     4.19   0.000     .3304544    .9109986
      sulphonylureas |   .1972307     .09528     2.07   0.038     .0104853     .383976
          biguanides |   .0025993   .0887686     0.03   0.977    -.1713839    .1765825
      other_diabetes |  -.5649218   .2838278    -1.99   0.047    -1.121214   -.0086296
        pioglitazone |  -.3193373   .2794181    -1.14   0.253    -.8669867    .2283121
       rosiglitazone |  -.1049629   .1710399    -0.61   0.539    -.4401948    .2302691
             CHD_365 |   1.491422   .1419413    10.51   0.000     1.213222    1.769622
      arrhythmia_365 |   .0716529   .3695892     0.19   0.846    -.6527285    .7960344
```

```
             HF_365 |   2.165542   .2301779     9.41   0.000     1.714402    2.616682
          renal_365 |   1.297418   .2644456     4.91   0.000      .779114    1.815722
   hypertension_365 |  -.4615257   .1141507    -4.04   0.000    -.6852569   -.2377945
         stroke_365 |   1.425298   .2379848     5.99   0.000     .9588564     1.89174
      neoplasms_365 |    2.72593   .1696337    16.07   0.000     2.393454    3.058406
            mal_365 |    .734511    .440781     1.67   0.096    -.1294038    1.598426
          liver_365 |   1.336851   .4724093     2.83   0.005     .4109461    2.262756
        lipid_d_365 |   -.604679   .0983019    -6.15   0.000    -.7973472   -.4120108
------------------------------------------------------------------------------------
```

-> agecat2 = 75

```
note: 2496 groups (2496 obs) dropped because of all positive or all negative outcomes.

Iteration 0:   log likelihood = -1558.2167
Iteration 1:   log likelihood = -1552.0386
Iteration 2:   log likelihood = -1552.0205
Iteration 3:   log likelihood = -1552.0205

Conditional (fixed-effects) logistic regression   Number of obs   =       6614
                                                  LR chi2(23)     =    1480.43
                                                  Prob > chi2     =     0.0000
Log likelihood = -1552.0205                       Pseudo R2       =     0.3229

------------------------------------------------------------------------------------
              _case |      Coef.   Std. Err.      z    P>|z|     [95% Conf. Interval]
--------------------+---------------------------------------------------------------
      range_hba1c365 |
                 0  |   .1705473   .0719242     2.37   0.018     .0295784    .3115162
                 2  |    .472212   .1189579     3.97   0.000     .2390589    .7053651
                    |
            smoking |
                 1  |   .2290639   .0717521     3.19   0.001     .0884324    .3696955
                 2  |   .5864219   .1090851     5.38   0.000      .372619    .8002247
                    |
range_bmi_365_simple |
                 2  |  -.5264533   .0748965    -7.03   0.000    -.6732477    -.379659
```

```
                3  |  -.4419398   .0841457    -5.25   0.000    -.6068624   -.2770172
                   |
     duration_diab |   .0165453   .0049929     3.31   0.001     .0067595    .0263312
          insulins |   .3137516   .1189637     2.64   0.008      .080587    .5469162
    sulphonylureas |   .1275802     .06776     1.88   0.060     -.005227    .2603874
        biguanides |  -.0943726   .0655016    -1.44   0.150    -.2227534    .0340082
    other_diabetes |  -.1313256   .2391345    -0.55   0.583    -.6000206    .3373693
      pioglitazone |  -.4722564   .2319839    -2.04   0.042    -.9269364   -.0175764
     rosiglitazone |  -.1948213    .141995    -1.37   0.170    -.4731264    .0834839
           CHD_365 |   1.290803   .1073795    12.02   0.000     1.080343    1.501263
    arrhythmia_365 |  -.0083389   .2207094    -0.04   0.970    -.4409213    .4242435
            HF_365 |   1.768976   .1466198    12.07   0.000     1.481607    2.056346
         renal_365 |   1.409199   .1647707     8.55   0.000     1.086254    1.732144
  hypertension_365 |  -.5108331   .0880819    -5.80   0.000    -.6834704   -.3381957
        stroke_365 |   1.846829   .1746917    10.57   0.000     1.504439    2.189218
     neoplasms_365 |   1.762024   .1072291    16.43   0.000     1.551859     1.97219
           mal_365 |   .1275953    .323925     0.39   0.694    -.5072859    .7624765
         liver_365 |   2.263535   .5639463     4.01   0.000     1.158221    3.368849
       lipid_d_365 |  -.5236589   .0703718    -7.44   0.000    -.6615851   -.3857327
------------------------------------------------------------------------------
```

-> agecat2 = 85

```
note: 1971 groups (1971 obs) dropped because of all positive or all negative outcomes.

Iteration 0:   log likelihood = -830.47279
Iteration 1:   log likelihood = -828.38752
Iteration 2:   log likelihood = -828.38427
Iteration 3:   log likelihood = -828.38427

Conditional (fixed-effects) logistic regression   Number of obs   =       3090
                                                  LR chi2(23)     =     485.06
                                                  Prob > chi2     =     0.0000
Log likelihood = -828.38427                       Pseudo R2       =     0.2265
```

_case	Coef.	Std. Err.	z	P>|z|	[95% Conf.	Interval]
range_hba1c365						
0	.0465476	.0956137	0.49	0.626	-.1408519	.233947
2	.2474235	.1661184	1.49	0.136	-.0781626	.5730096
smoking						
1	.0501812	.0935036	0.54	0.591	-.1330825	.2334449
2	.2335793	.1997027	1.17	0.242	-.1578308	.6249895
range_bmi_365_simple						
2	-.3928797	.0960522	-4.09	0.000	-.5811385	-.2046208
3	-.4251726	.1238971	-3.43	0.001	-.6680066	-.1823387
duration_diab	.0138341	.0061823	2.24	0.025	.001717	.0259512
insulins	.0153424	.1748608	0.09	0.930	-.3273786	.3580633
sulphonylureas	.0403058	.0888714	0.45	0.650	-.133879	.2144906
biguanides	.0628447	.0954676	0.66	0.510	-.1242684	.2499577
other_diabetes	.514455	.4519614	1.14	0.255	-.3713731	1.400283
pioglitazone	.7813174	.4678069	1.67	0.095	-.1355672	1.698202
rosiglitazone	.0457404	.2322761	0.20	0.844	-.4095125	.5009932
CHD_365	1.147999	.1581416	7.26	0.000	.8380472	1.457951
arrhythmia_365	-.5469572	.2882008	-1.90	0.058	-1.11182	.017906
HF_365	1.367136	.1786897	7.65	0.000	1.01691	1.717361
renal_365	.9778866	.2096038	4.67	0.000	.5670707	1.388703
hypertension_365	-.4658408	.1367825	-3.41	0.001	-.7339296	-.1977521
stroke_365	1.861267	.2251901	8.27	0.000	1.419902	2.302631
neoplasms_365	1.15788	.1509016	7.67	0.000	.8621183	1.453642
mal_365	.5707671	.4284688	1.33	0.183	-.2690164	1.410551
liver_365	2.222941	1.131858	1.96	0.050	.0045412	4.441341
lipid_d_365	-.6640567	.0975502	-6.81	0.000	-.8552517	-.4728618

Table 1: Association between mortality and HbA1c

MULTIPLE IMPUTATION ANALYSIS

MODEL 1 (unadjusted): HbA1c <6.5% (coded 0), HbA1c>9.0% (coded 2) versus base HbA1c >=6.5% & <=9.0% (coded 1)

```
. mi estimate: clogit _c ib1.range_hba1c1, group(_set) or

Multiple-imputation estimates                   Imputations     =         10
Conditional (fixed-effects) logistic regression Number of obs   =      33170
                                                Average RVI     =     0.3377
                                                Largest FMI     =     0.2827
DF adjustment:   Large sample                   DF:     min     =     122.50
                                                        avg     =     185.45
                                                        max     =     248.40
Model F test:       Equal FMI                   F(   2,  188.7) =      35.42
Within VCE type:          OIM                   Prob > F        =     0.0000

------------------------------------------------------------------------------
       _case |      Coef.   Std. Err.      t    P>|t|     [95% Conf. Interval]
-------------+----------------------------------------------------------------
range_hba1c1 |
          0  |   .1304331   .0270598     4.82   0.000     .0771372     .183729
          2  |   .3387023    .043372     7.81   0.000     .2528466    .4245579
------------------------------------------------------------------------------
```

Table 1: Association between mortality and HbA1c

MULTIPLE IMPUTATION ANALYSIS

MODEL 1 (adjusted): HbA1c <6.5% (coded 0), HbA1c>9.0% (coded 2) versus base HbA1c >=6.5% & <=9.0% (coded 1)

```
. #delimit ;
delimiter now ;
. mi estimate: clogit _c ib1.range_hba1c1
>          i.smoking
>          i.range_bmi_365_simple
>          duration_diab
>          insulins sulphonylureas biguanides other_diabetes pioglitazone rosiglitazone
>          CHD_365 arrhythmia_365 HF_365 renal_365 hypertension_365 stroke_365 neoplasms_365 mal_365 liver_365
>      lipid_d_365
>          , group(_set)
> ;

Multiple-imputation estimates                    Imputations     =         10
Conditional (fixed-effects) logistic regression  Number of obs   =      33170
                                                 Average RVI     =     0.0492
                                                 Largest FMI     =     0.2849
DF adjustment:   Large sample                    DF:     min     =     120.66
                                                         avg     = 1217579.29
                                                         max     =   1.37e+07
Model F test:       Equal FMI                    F(  23,90643.4) =     148.67
Within VCE type:          OIM                    Prob > F        =     0.0000

------------------------------------------------------------------------------
               _case |      Coef.   Std. Err.      t    P>|t|     [95% Conf. Interval]
---------------------+--------------------------------------------------------
        range_hba1c1 |
                  0  |   .1124487    .035102     3.20   0.002     .0432143     .181683
                  2  |   .2564888   .0543022     4.72   0.000     .1489801    .3639974
                     |
             smoking |
                  1  |   .1039872   .0314263     3.31   0.001     .0423844    .1655901
```

2	.4854813	.0446877	10.86	0.000	.3978894	.5730731
range_bmi_365_simple						
2	-.4491288	.0343714	-13.07	0.000	-.5165685	-.3816892
3	-.3970566	.0390311	-10.17	0.000	-.4737107	-.3204024
duration_diab	.0116412	.0022048	5.28	0.000	.0073199	.0159626
insulins	.3481333	.0530264	6.57	0.000	.2441863	.4520803
sulphonylureas	.1935541	.0295578	6.55	0.000	.135619	.2514892
biguanides	-.1282333	.0302782	-4.24	0.000	-.1875788	-.0688878
other_diabetes	-.1267919	.1128412	-1.12	0.261	-.3479573	.0943735
pioglitazone	-.3374105	.1209232	-2.79	0.005	-.5744156	-.1004054
rosiglitazone	-.1367892	.0734568	-1.86	0.063	-.2807619	.0071835
CHD_365	1.107754	.0474544	23.34	0.000	1.014745	1.200763
arrhythmia_365	-.0143922	.1054018	-0.14	0.891	-.2209762	.1921917
HF_365	1.512098	.063478	23.82	0.000	1.387683	1.636513
renal_365	1.219919	.080072	15.24	0.000	1.06298	1.376859
hypertension_365	-.6299584	.0413657	-15.23	0.000	-.7110339	-.548883
stroke_365	1.682953	.0728388	23.11	0.000	1.540191	1.825715
neoplasms_365	1.754597	.0486523	36.06	0.000	1.65924	1.849954
mal_365	.443128	.1546697	2.86	0.004	.1399803	.7462757
liver_365	1.945107	.2106871	9.23	0.000	1.532167	2.358047
lipid_d_365	-.6043242	.0321914	-18.77	0.000	-.6674183	-.5412302

Table 1: Association between mortality and HbA1c

MULTIPLE IMPUTATION ANALYSIS

MODEL 2 (unadjusted):
  HbA1c <6.5% (coded 0), HbA1c>9.0% (coded 2) versus base HbA1c >=6.5% & <=9.0% (coded 1)
  Change HbA1c <1% (coded 0), change HbA1c>1.0% (coded 2) versus base change HbA1c >=1% & <=1% (coded 1)

```
.
. #delimit ;
delimiter now ;
. mi estimate: clogit _c
>   ib1.range_hba1c1
>   ib1.change_cat365
>         , group(_set)
> ;

Multiple-imputation estimates                   Imputations     =         10
Conditional (fixed-effects) logistic regression   Number of obs   =      33170
                                                Average RVI     =     0.6482
                                                Largest FMI     =     0.5404
DF adjustment:   Large sample                   DF:     min     =      34.05
                                                        avg     =     131.90
                                                        max     =     319.03
Model F test:       Equal FMI                   F(   4,  197.2) =      24.21
Within VCE type:          OIM                   Prob > F        =     0.0000

------------------------------------------------------------------------------
       _case |      Coef.   Std. Err.      t    P>|t|     [95% Conf. Interval]
-------------+----------------------------------------------------------------
 range_hba1c1 |
          0  |   .1453875   .0268738     5.41   0.000     .0925152    .1982598
          2  |   .2665351   .0462837     5.76   0.000     .1749736    .3580967
             |
change_cat365 |
          0  |   .2453228   .0621554     3.95   0.000     .1200343    .3706113
          2  |   .2239997   .0477103     4.69   0.000     .1270456    .3209538
------------------------------------------------------------------------------
```

Table 1: Association between mortality and HbA1c

MULTIPLE IMPUTATION ANALYSIS

```
MODEL 2 (adjusted):
  HbA1c <6.5% (coded 0), HbA1c>9.0% (coded 2) versus base HbA1c >=6.5% & <=9.0% (coded 1)
  Change HbA1c <1% (coded 0),  change HbA1c>1.0% (coded 2) versus base change HbA1c >=1% & <=1% (coded 1)


. #delimit cr
delimiter now cr
.
.
. #delimit ;
delimiter now ;
. mi estimate: clogit _c
>   ib1.range_hba1c1
>   ib1.change_cat
>         i.smoking
>         i.range_bmi_365_simple
>         duration_diab
>         insulins sulphonylureas biguanides other_diabetes pioglitazone rosiglitazone
>         CHD_365 arrhythmia_365 HF_365 renal_365 hypertension_365 stroke_365 neoplasms_365 mal_365 liver_365
>     lipid_d_365
>         , group(_set)
> ;


Multiple-imputation estimates                   Imputations     =         10
Conditional (fixed-effects) logistic regression   Number of obs   =      33170
                                                Average RVI     =     0.1355
                                                Largest FMI     =     0.6041
DF adjustment:   Large sample                   DF:     min     =      27.13
                                                        avg     =  554051.82
                                                        max     = 4302563.97
Model F test:       Equal FMI                   F(  25,15280.6) =     126.63
Within VCE type:          OIM                   Prob > F        =     0.0000
```

_case	Coef.	Std. Err.	t	P>|t|	[95% Conf.	Interval]
range_hba1c1						
0	.120761	.0356449	3.39	0.001	.0503916	.1911303
2	.2041723	.0577185	3.54	0.001	.0899665	.318378
change_cat365						
0	.1914302	.086612	2.21	0.036	.0137568	.3691035
2	.1472914	.0572591	2.57	0.014	.0314016	.2631811
smoking						
1	.105162	.0315254	3.34	0.001	.0433623	.1669617
2	.482715	.0446332	10.82	0.000	.3952319	.570198
range_bmi_365_simple						
2	-.448057	.0342195	-13.09	0.000	-.5151838	-.3809302
3	-.3947886	.0391289	-10.09	0.000	-.471642	-.3179352
duration_diab	.0116833	.0022086	5.29	0.000	.0073544	.0160121
insulins	.3309788	.0536085	6.17	0.000	.2258813	.4360764
sulphonylureas	.1852324	.0297507	6.23	0.000	.1269171	.2435477
biguanides	-.1343875	.030616	-4.39	0.000	-.1943987	-.0743764
other_diabetes	-.1321591	.1129288	-1.17	0.242	-.3534958	.0891775
pioglitazone	-.345329	.1210738	-2.85	0.004	-.5826295	-.1080286
rosiglitazone	-.1388627	.0736679	-1.88	0.059	-.2832496	.0055242
CHD_365	1.110058	.047564	23.34	0.000	1.016834	1.203283
arrhythmia_365	-.0116876	.1054881	-0.11	0.912	-.2184405	.1950654
HF_365	1.51012	.0635774	23.75	0.000	1.38551	1.634729
renal_365	1.218898	.0801664	15.20	0.000	1.061773	1.376023
hypertension_365	-.6294875	.0414065	-15.20	0.000	-.7106428	-.5483322
stroke_365	1.682633	.0728371	23.10	0.000	1.539875	1.825391
neoplasms_365	1.752987	.0487684	35.95	0.000	1.657402	1.848572
mal_365	.4309238	.154964	2.78	0.005	.1271984	.7346492
liver_365	1.943616	.2110337	9.21	0.000	1.529997	2.357236
lipid_d_365	-.6030374	.032236	-18.71	0.000	-.6662188	-.5398559

Table 2. Association between mortality and HbA1c, stratified by age group

MULTIPLE IMPUTATION ANALYSIS

Unadjusted: HbA1c <6.5% (coded 0), HbA1c>9.0% (coded 2) versus base HbA1c >=6.5% & <=9.0% (coded 1)

```
note:
Agecat2
 45 "<55"
 55 "55-64"
 65 "65-74"
 75 "75-84"
 85 "85+"
.
. foreach agecat in 45 55 65 75 85 {
  2. noisily disp "AGECAT2 = `agecat'"
  3. noisily disp ""
  4. mi estimate: clogit _c ib1.range_hba1c1 if agecat2==`agecat', group(_set) or
  5. }
```

AGECAT2 = 45

```
Multiple-imputation estimates                   Imputations     =         10
Conditional (fixed-effects) logistic regression  Number of obs   =        864
                                                 Average RVI     =     0.2500
                                                 Largest FMI     =     0.2012
DF adjustment:   Large sample                    DF:     min     =     237.92
                                                         avg     =     249.00
                                                         max     =     260.08
Model F test:       Equal FMI                    F(   2,  294.6) =       4.34
Within VCE type:          OIM                    Prob > F        =     0.0138
------------------------------------------------------------------------------
       _case |      Coef.   Std. Err.      t    P>|t|     [95% Conf. Interval]
-------------+----------------------------------------------------------------
range_hba1c1 |
          0  |   .5310569    .182101     2.92   0.004     .1724768    .8896371
          2  |   .2906949   .2048314     1.42   0.157    -.1128198    .6942097
------------------------------------------------------------------------------
```

AGECAT2 = 55

```
Multiple-imputation estimates                     Imputations     =         10
Conditional (fixed-effects) logistic regression   Number of obs   =       2756
                                                  Average RVI     =     0.1761
                                                  Largest FMI     =     0.1750
DF adjustment:   Large sample                     DF:     min     =     312.40
                                                          avg     =     444.01
                                                          max     =     575.62
Model F test:       Equal FMI                     F(   2,  515.8) =       8.11
Within VCE type:          OIM                     Prob > F        =     0.0003

------------------------------------------------------------------------------
       _case |      Coef.   Std. Err.      t    P>|t|     [95% Conf. Interval]
-------------+----------------------------------------------------------------
range_hba1c1 |
          0  |   .2808729   .0970119     2.90   0.004     .0903324    .4714134
          2  |   .3968415   .1159602     3.42   0.001     .1686798    .6250032
------------------------------------------------------------------------------
```

AGECAT2 = 65

```
Multiple-imputation estimates                     Imputations     =         10
Conditional (fixed-effects) logistic regression   Number of obs   =       7684
                                                  Average RVI     =     0.1737
                                                  Largest FMI     =     0.1948
DF adjustment:   Large sample                     DF:     min     =     253.41
                                                          avg     =     434.11
                                                          max     =     614.81
Model F test:       Equal FMI                     F(   2,  528.0) =      18.04
Within VCE type:          OIM                     Prob > F        =     0.0000
------------------------------------------------------------------------------
       _case |      Coef.   Std. Err.      t    P>|t|     [95% Conf. Interval]
-------------+----------------------------------------------------------------
range_hba1c1 |
          0  |   .1501177   .0582448     2.58   0.011     .0354122    .2648233
          2  |   .4706783   .0788655     5.97   0.000     .3157999    .6255568
------------------------------------------------------------------------------
```

AGECAT2 = 75

```
Multiple-imputation estimates                     Imputations     =         10
Conditional (fixed-effects) logistic regression   Number of obs   =      12992
                                                  Average RVI     =     0.2613
                                                  Largest FMI     =     0.2099
DF adjustment:   Large sample                     DF:     min     =     219.09
                                                          avg     =     301.43
                                                          max     =     383.77
Model F test:       Equal FMI                     F(   2,  275.3) =      13.11
Within VCE type:          OIM                     Prob > F        =     0.0000

------------------------------------------------------------------------------
       _case |      Coef.   Std. Err.      t    P>|t|     [95% Conf. Interval]
-------------+----------------------------------------------------------------
range_hba1c1 |
          0  |   .1072934   .0417106     2.57   0.010     .0252835    .1893033
          2  |   .3438707   .0699169     4.92   0.000     .2060749    .4816664
------------------------------------------------------------------------------
```

AGECAT2 = 85

```
Multiple-imputation estimates                     Imputations     =         10
Conditional (fixed-effects) logistic regression   Number of obs   =       8874
                                                  Average RVI     =     0.2915
                                                  Largest FMI     =     0.2147
DF adjustment:   Large sample                     DF:     min     =     209.47
                                                          avg     =     222.95
                                                          max     =     236.42
Model F test:       Equal FMI                     F(   2,  233.6) =       2.61
Within VCE type:          OIM                     Prob > F        =     0.0758
------------------------------------------------------------------------------
       _case |      Coef.   Std. Err.      t    P>|t|     [95% Conf. Interval]
-------------+----------------------------------------------------------------
range_hba1c1 |
          0  |   .0749961   .0507421     1.48   0.141    -.0249683    .1749606
          2  |   .1814217   .0871325     2.08   0.039     .0096527    .3531907
------------------------------------------------------------------------------
```

Table 2. Association between mortality and HbA1c, stratified by age group

MULTIPLE IMPUTATION ANALYSIS

Adjusted: HbA1c <6.5% (coded 0), HbA1c>9.0% (coded 2) versus base HbA1c >=6.5% & <=9.0% (coded 1)

```
note:
Agecat2
 45 "<55"
 55 "55-64"
 65 "65-74"
 75 "75-84"
 85 "85+"

. foreach agecat in 45 55 65 75 85 {
  2. noisily disp "AGECAT2 = `agecat'"
  3. noisily disp ""
  4. #delimit ;
delimiter now ;
. mi estimate: clogit _c ib1.range_hba1c1
>         i.smoking
>         i.range_bmi_365_simple
>         duration_diab
>         insulins sulphonylureas biguanides other_diabetes pioglitazone rosiglitazone
>         CHD_365 arrhythmia_365 HF_365 renal_365 hypertension_365 stroke_365 neoplasms_365 mal_365 liver_365
>     lipid_d_365
>           if agecat2==`agecat'
>         , group(_set)
> ;
  5. #delimit cr
delimiter now cr
. }
AGECAT2 = 45

Multiple-imputation estimates                    Imputations     =         10
Conditional (fixed-effects) logistic regression   Number of obs   =        864
                                                  Average RVI     =     0.0486
                                                  Largest FMI     =     0.3515
```

```
DF adjustment:   Large sample                     DF:     min     =      79.95
                                                          avg     =   1.68e+12
                                                          max     =   2.74e+13
Model F test:       Equal FMI                     F(  23,92631.8) =       3.48
Within VCE type:          OIM                     Prob > F        =     0.0000
------------------------------------------------------------------------------
               _case |      Coef.   Std. Err.      t    P>|t|     [95% Conf. Interval]
---------------------+--------------------------------------------------------
        range_hba1c1 |
                  0  |   .4270822   .3011707     1.42   0.160    -.1722722    1.026437
                  2  |   .1864773   .2755489     0.68   0.499    -.3542234     .727178
                     |
             smoking |
                  1  |  -.0495322   .2575567    -0.19   0.847      -.55438    .4553157
                  2  |    .843486   .2364531     3.57   0.000      .379782     1.30719
                     |
range_bmi_365_simple |
                  2  |  -.4590229   .3089758    -1.49   0.138    -1.065159    .1471135
                  3  |  -.1276192   .2870036    -0.44   0.657    -.6904922    .4352538
                     |
       duration_diab |   .0113298   .0260695     0.43   0.664    -.0397692    .0624288
            insulins |   .6585507    .361515     1.82   0.069    -.0501278    1.367229
      sulphonylureas |  -.1955548   .2215439    -0.88   0.377     -.629898    .2387884
         biguanides  |  -.4497186    .210756    -2.13   0.033    -.8628336   -.0366037
      other_diabetes |   .1285425   1.107575     0.12   0.908    -2.042292    2.299377
        pioglitazone |  -.5470899   .6585057    -0.83   0.406    -1.837745    .7435655
       rosiglitazone |  -.8334389   .5512882    -1.51   0.131    -1.913946    .2470682
             CHD_365 |   1.162546   .3985275     2.92   0.004     .3814242    1.943667
      arrhythmia_365 |   .0624738   1.039861     0.06   0.952    -1.975645    2.100593
              HF_365 |   26.16718   1778.142     0.01   0.988    -3458.928    3511.262
           renal_365 |   1.431623   .8874198     1.61   0.107    -.3077146     3.17096
    hypertension_365 |  -.0534747   .3384997    -0.16   0.874    -.7169262    .6099767
          stroke_365 |    3.25175   1.230091     2.64   0.008     .8407008      5.6628
       neoplasms_365 |    2.83246   .4306965     6.58   0.000     1.988306    3.676614
             mal_365 |   15.41925    1254.96     0.01   0.990    -2444.257    2475.095
           liver_365 |   3.748761   1.216755     3.08   0.002     1.363911    6.133612
         lipid_d_365 |  -.1561007   .2062536    -0.76   0.449    -.5603578    .2481564
------------------------------------------------------------------------------
```

AGECAT2 = 55

```
Multiple-imputation estimates                      Imputations     =         10
Conditional (fixed-effects) logistic regression   Number of obs   =       2756
                                                   Average RVI     =     0.0448
                                                   Largest FMI     =     0.2745
DF adjustment:   Large sample                      DF:     min     =     129.73
                                                           avg     =  190411.36
                                                           max     =  793787.49
Model F test:       Equal FMI                      F(  23,108540.4)=      13.02
Within VCE type:          OIM                      Prob > F        =     0.0000
```

_case	Coef.	Std. Err.	t	P>|t|	[95% Conf.	Interval]
range_hba1c1						
0	.3481298	.1553744	2.24	0.026	.041771	.6544886
2	.4930847	.1887061	2.61	0.010	.119745	.8664245
smoking						
1	.4771374	.1362289	3.50	0.000	.2100754	.7441994
2	.9379886	.1637884	5.73	0.000	.6167217	1.259255
range_bmi_365_simple						
2	-.6548932	.1796479	-3.65	0.000	-1.007281	-.302505
3	-.3846209	.1732681	-2.22	0.027	-.7244269	-.0448148
duration_diab	.0502395	.0120468	4.17	0.000	.0266281	.073851
insulins	.2957841	.1906023	1.55	0.121	-.0778068	.6693749
sulphonylureas	.3348157	.1292947	2.59	0.010	.0813822	.5882493
biguanides	-.3848292	.1248493	-3.08	0.002	-.6295349	-.1401234
other_diabetes	-.6460408	.3839138	-1.68	0.092	-1.3985	.1064182
pioglitazone	-.1357406	.3982483	-0.34	0.733	-.916297	.6448159
rosiglitazone	-.2488197	.2717466	-0.92	0.360	-.7814365	.2837971
CHD_365	1.449108	.1937915	7.48	0.000	1.069279	1.828938
arrhythmia_365	.2517192	.5042894	0.50	0.618	-.7366719	1.24011
HF_365	2.676696	.4471453	5.99	0.000	1.800303	3.553089
renal_365	2.275757	.5183791	4.39	0.000	1.259751	3.291763

```
    hypertension_365 |  -.4875328   .1625072    -3.00   0.003    -.8060423   -.1690232
          stroke_365 |     1.8562   .3950323     4.70   0.000     1.081947    2.630453
       neoplasms_365 |    3.31004   .2634398    12.56   0.000     2.793705    3.826374
             mal_365 |   2.011608   .8217744     2.45   0.014     .4008648    3.622351
           liver_365 |   2.549885   .5307188     4.80   0.000     1.509688    3.590082
         lipid_d_365 |  -.5816513   .1261706    -4.61   0.000    -.8289424   -.3343602
--------------------------------------------------------------------------------------
```

AGECAT2 = 65

```
Multiple-imputation estimates                     Imputations     =         10
Conditional (fixed-effects) logistic regression   Number of obs   =       7684
                                                  Average RVI     =     0.0344
                                                  Largest FMI     =     0.2762
DF adjustment:   Large sample                     DF:     min     =     128.12
                                                          avg     =  878819.30
                                                          max     = 4723056.78
Model F test:       Equal FMI                    F(  23,180093.5)=      38.38
Within VCE type:          OIM                     Prob > F        =     0.0000

--------------------------------------------------------------------------------------
               _case |      Coef.   Std. Err.      t    P>|t|     [95% Conf. Interval]
---------------------+----------------------------------------------------------------
        range_hba1c1 |
                  0  |   .1763825    .085246     2.07   0.041     .0077104    .3450547
                  2  |    .381604   .1061233     3.60   0.000      .173318      .58989
                     |
             smoking |
                  1  |   .2484003   .0720732     3.45   0.001     .1071041    .3896965
                  2  |    .510348   .0926159     5.51   0.000     .3287447    .6919514
                     |
range_bmi_365_simple |
                  2  |   -.520852   .0856397    -6.08   0.000    -.6888112   -.3528928
                  3  |  -.4574075   .0862556    -5.30   0.000    -.6265441   -.2882709
                     |
       duration_diab |   .0158543   .0054324     2.92   0.004      .005207    .0265017
            insulins |   .5870884   .1117118     5.26   0.000     .3681066    .8060702
```

```
     sulphonylureas |   .2956492   .0686462     4.31   0.000     .1611033     .430195
         biguanides |  -.1249549   .0670957    -1.86   0.063    -.2564659    .0065561
     other_diabetes |  -.3466001    .216842    -1.60   0.110     -.771603    .0784029
       pioglitazone |  -.4098541   .2393476    -1.71   0.087    -.8789675    .0592593
      rosiglitazone |  -.1025182   .1446306    -0.71   0.478    -.3859891    .1809527
            CHD_365 |   1.281901   .1034171    12.40   0.000     1.079207    1.484595
     arrhythmia_365 |    .134905   .2684789     0.50   0.615    -.3913054    .6611154
             HF_365 |   2.126002   .1694927    12.54   0.000     1.793803    2.458202
          renal_365 |   1.513705   .1975549     7.66   0.000     1.126503    1.900906
   hypertension_365 |  -.5954141   .0872907    -6.82   0.000    -.7665018   -.4243264
         stroke_365 |   1.671551   .1870792     8.93   0.000     1.304883     2.03822
      neoplasms_365 |    2.47221   .1169795    21.13   0.000     2.242935    2.701486
            mal_365 |   .5934294   .3601096     1.65   0.099    -.1123732    1.299232
          liver_365 |   1.371757   .3595508     3.82   0.000       .66705    2.076464
        lipid_d_365 |  -.5926382   .0698561    -8.48   0.000    -.7295539   -.4557224
------------------------------------------------------------------------------------
```

AGECAT2 = 75

```
Multiple-imputation estimates                    Imputations     =         10
Conditional (fixed-effects) logistic regression  Number of obs   =      12992
                                                 Average RVI     =     0.0552
                                                 Largest FMI     =     0.2947
DF adjustment:   Large sample                    DF:     min     =     112.91
                                                         avg     = 1769414.29
                                                         max     =   1.99e+07
Model F test:       Equal FMI                    F(  23,72915.9) =      58.83
Within VCE type:          OIM                    Prob > F        =     0.0000


------------------------------------------------------------------------------------
              _case |      Coef.   Std. Err.      t    P>|t|     [95% Conf. Interval]
--------------------+---------------------------------------------------------------
        range_hba1c1 |
                  0  |   .0791185   .0581259     1.36   0.176    -.0360402    .1942773
                  2  |   .2533065   .0882368     2.87   0.005     .0792007    .4274123
                     |
```

```
             smoking |
                  1  |   .1106534   .0514603     2.15   0.032     .0096672    .2116396
                  2  |   .5200147   .0759349     6.85   0.000     .3710405    .6689889
                     |
range_bmi_365_simple |
                  2  |  -.4481933   .0554492    -8.08   0.000    -.5571991   -.3391875
                  3  |  -.4106362   .0600808    -6.83   0.000    -.5285042   -.2927682
                     |
       duration_diab |   .0122438   .0035143     3.48   0.000     .0053558    .0191318
            insulins |   .3926144   .0868345     4.52   0.000     .2223759    .5628529
      sulphonylureas |   .1981589   .0476079     4.16   0.000     .1048409     .291477
          biguanides |  -.1566047    .047652    -3.29   0.001    -.2500019   -.0632074
      other_diabetes |  -.0807279   .1793353    -0.45   0.653    -.4322197    .2707639
        pioglitazone |  -.4521031   .1937256    -2.33   0.020    -.8317983   -.0724078
       rosiglitazone |  -.1514568   .1162917    -1.30   0.193    -.3793844    .0764709
             CHD_365 |    1.08934   .0731545    14.89   0.000     .9459596    1.232721
      arrhythmia_365 |  -.0094964   .1562943    -0.06   0.952    -.3158278    .2968349
              HF_365 |   1.431551   .0966469    14.81   0.000     1.242127    1.620976
           renal_365 |   1.199461   .1206346     9.94   0.000     .9630209    1.435901
    hypertension_365 |  -.6041807   .0638129    -9.47   0.000    -.7292518   -.4791096
          stroke_365 |    1.71522   .1122841    15.28   0.000     1.495147    1.935293
       neoplasms_365 |   1.657752   .0752188    22.04   0.000     1.510326    1.805178
             mal_365 |   .1816836   .2433511     0.75   0.455    -.2952798     .658647
           liver_365 |   1.795616   .3845792     4.67   0.000     1.041854    2.549378
         lipid_d_365 |  -.6627958   .0505122   -13.12   0.000     -.761798   -.5637936
--------------------------------------------------------------------------------------
```

AGECAT2 = 85

```
Multiple-imputation estimates                    Imputations     =         10
Conditional (fixed-effects) logistic regression  Number of obs   =       8874
                                                 Average RVI     =     0.0762
                                                 Largest FMI     =     0.2631
DF adjustment:   Large sample                    DF:     min     =     140.91
                                                         avg     = 1311900.20
                                                         max     =   2.32e+07
```

```
Model F test:       Equal FMI                    F(  23,39793.5) =      29.46
Within VCE type:          OIM                    Prob > F        =     0.0000
```

_case	Coef.	Std. Err.	t	P>|t|	[95% Conf.	Interval]
range_hba1c1						
0	.0366487	.0600644	0.61	0.542	-.081671	.1549684
2	.1381378	.1034876	1.33	0.184	-.0664513	.3427268
smoking						
1	-.0534786	.0606408	-0.88	0.378	-.172765	.0658078
2	.2433843	.1099262	2.21	0.027	.0274756	.4592931
range_bmi_365_simple						
2	-.3691392	.060262	-6.13	0.000	-.4878336	-.2504448
3	-.4030307	.0798716	-5.05	0.000	-.560522	-.2455395
duration_diab	.0048216	.003517	1.37	0.170	-.0020716	.0117147
insulins	-.1444727	.1116268	-1.29	0.196	-.3632913	.074346
sulphonylureas	.1046128	.0517153	2.02	0.043	.0032361	.2059895
biguanides	-.0147625	.05856	-0.25	0.801	-.1295455	.1000205
other_diabetes	.3172699	.2760103	1.15	0.250	-.2237028	.8582425
pioglitazone	.0778027	.3073615	0.25	0.800	-.5246157	.680221
rosiglitazone	.0415552	.1655783	0.25	0.802	-.2829741	.3660845
CHD_365	.9259599	.0923119	10.03	0.000	.7450307	1.106889
arrhythmia_365	-.1465989	.1865521	-0.79	0.432	-.5122357	.2190379
HF_365	1.213739	.1017742	11.93	0.000	1.014265	1.413213
renal_365	.9682997	.1348157	7.18	0.000	.7040651	1.232534
hypertension_365	-.7446289	.085559	-8.70	0.000	-.9123219	-.5769359
stroke_365	1.581232	.1188263	13.31	0.000	1.348335	1.81413
neoplasms_365	.9511703	.089411	10.64	0.000	.775927	1.126414
mal_365	.3720135	.2636224	1.41	0.158	-.144677	.8887041
liver_365	2.382935	.7879871	3.02	0.002	.8385084	3.927361
lipid_d_365	-.6138135	.0647455	-9.48	0.000	-.7407128	-.4869142
